# Determinants for not utilizing trachomatous trichiasis surgery among trachomatous trichiasis patients in Mehalsayint District, North-East Ethiopia

**DOI:** 10.1371/journal.pntd.0006669

**Published:** 2018-07-18

**Authors:** Tedla Desta Meshesha, Goitom Halefom Senbete, Getahun Gebre Bogale

**Affiliations:** 1 Trachoma Program at East Amhara Sub-Regional Office, The Carter Center, Dessie, Ethiopia; 2 Department of Public Health, College of Medicine and Health Science, Wollo University, Dessie, Ethiopia; 3 Department of Health Information Technology, Dessie Health Science College, Dessie, Ethiopia; University of California San Diego School of Medicine, UNITED STATES

## Abstract

**Background:**

Globally, trachoma is the leading cause of infectious blindness. In Ethiopia, the overall Trachomatous Trichiasis (TT) surgical coverage is 41%. Identifying determinants for not utilizing TT surgery among TT patients is important to design and monitor effective intervention programs. Therefore, this study aimed to identify determinants for not utilizing TT surgery among TT patients in Mehalsayint District, North East Ethiopia.

**Methodology/Principal findings:**

A community based unmatched case control study was employed from March 30, 2017 to April 13, 2017. A total of 482 study participants (241 cases and 241 controls) with age of ≥15 years were included in the study. The data were entered with Epi info version 7.2 software and exported to SPSS version 20 for analysis. Bivariate analysis was fitted to screen candidate variables with *p<*0.2 for the final model. Finally, multivariable logistic regression analysis was employed to identify significant factors (*p<*0.05) for not utilizing TT surgery. Respondents’ age of 16–30 years (AOR: 10.11; 95% CI: 2.72, 37.59) and widowed respondents (AOR: 0.40; 95% CI: 0.21, 0.77), time to reach the service (AOR: 0.46; 95% CI: 0.24, 0.87), unavailability of TT surgeon (AOR: 5.00; 95% CI: 1.16, 21.38), symptoms of trichiasis (AOR: 7.49; 95% CI: 2.41, 23.26), duration of the problem (AOR: 2.56; 95% CI: 1.44, 4.54), the affected eye (AOR: 2.16; 95% CI: 1.23, 3.80), epilation practice (AOR: 3.22; 95% CI: 1.84, 5.64), and place of TT surgery given (AOR: 4.21; 95% CI: 2.48, 7.14) were significant determinants for not utilizing TT surgical services.

**Conclusions/Significance:**

In this study, TT surgery against trachoma is very low and TT remains public health problem in the district. Being younger age and widowed, time taken to reach the service, absence of TT surgeon, symptoms of trichiasis, duration of problem, the affected eye, epilation practice, and service place were determinants for the inability of TT surgical services. The findings of this study would help in designing effective interventions to reduce trachoma in that district.

## Author summary

Trachoma is the common ophthalmic infection and cause of blindness worldwide. It is caused by ocular infections with causative agent of *Chlamydia trachomatis* that might effect in chronic inflammation of the eyelids, which produces scarring of the conjunctiva that can consequently cause entropion trichiasis, resulting in interned eyelashes. The interned eyelashes as well as other changes of the eye, harm the cornea causing severe pain, corneal opacity and resulting vision loss. Over a million people in Ethiopia are estimated to have Trachomatous trichiasis (TT). Trachomatous trichiasis surgery is the backbone treatment option. Though the provision of free surgical services in the country exists, utilization rates are very low. Identifying the determinants for not utilizing the service is mandatory to take measures towards surgical uptake. A total of 482 study participants (241 cases and 241 controls) with age of ≥15 years were included in the study. The determinants for not use of surgical services were respondents in the younger age group (16–30 years) and widowed participants, lengthy distance from the service, unavailability of TT surgeon, no trichiasis symptoms, long time knowing the problem, right/left eye affected, no experience of epilation practice, and participants who knew place of service was given at health center.

## Introduction

Globally, trachoma is the leading cause of infectious blindness of the eye [1]. It is caused by *Chlamydia trachomatis* that might effect in chronic inflammation of the eyelids. Based on WHO grading system for trachoma, the disease classifies into five grades. These are Trachomatous Inflammation-Follicular (TF), which mostly requires topical treatment; Trachomatous Inflammation-Intense (TI), which topical and systemic treatments are considered; Trachomatous Scarring (TS)-when scars are visible as in the tarsal conjunctiva and which may obscure tarsal blood vessels; Trachomatous Trichiasis (TT)-when an individual is referred for eyelid surgery; and Corneal Opacity-a stage during which a person is irreversibly blind [2]. At the third grade, the eyelids inflammation creates scarring of the conjunctiva that can consequently cause entropion trichiasis, resulting in interned eyelashes. The jailed eyelashes as well as other changes of the eye, such as corneal limbus and lacrimal function, harm the cornea causing severe pain, opacity of cornea and resulting vision loss [3]. Epidemiologically, worldwide, there are about 146 million active cases, 10.6 million with trichiasis and 5.9 million blind. Twenty-seven million cases of active trachoma and 3.8 million cases of trichiasis are found in 28 of the 46 countries in the WHO African Region, with 279 million estimated number population living in endemic areas [1]. In Ethiopia, around 1.2 million people are blind; about 2.7 million people are living with low vision; over 9 million children are affected by trachoma; and over 1.2 million adults are suffering from trachomatous trichiasis [4].

Low uptake of TT surgery has always been a concern for the success of the surgical services in trachoma-control strategy. Awkwardly, surgical coverage in affected communities of Africa is lower than 50% [5]. A review paper of Trichiasis surgical coverage in sub-Saharan Africa indicated that there was a fivefold variation between the lowest coverage (9%) and the highest coverage (55%); the mean was 30.4% (±SD 11.6%) [6]. In Ethiopia, the overall coverage of trichiasis surgery was 41% [7]. The surgical treatment of trachomatous trichiasis is provided for free or subsidized in most trachoma endemic settings like Ethiopia. However, only 18–66% of TT patients attend for the service [8].

Various studies have examined why people with TT do not access the surgical services. Some of the reported causes of poor uptake of surgical services were unaware of how to access services, fear of surgery, burden of household tasks, indirect cost of surgery, longer walking distance, sex, family size, doubt surrounding the outcome, and absence of a companion and mildness of the condition [8–12].

To control and prevent this problem, international strategy had been created to eliminate trachoma as a blinding disease. The WHO-developed strategy is a combination of interventions known by ‘SAFE”. SAFE—stands for surgery for trichiasis (interned eyelashes), antibiotics, facial cleanliness and environmental improvement [13]. In Ethiopia, by sharing the strategy, the national blindness and trachoma program was initiated in 1976. Vision 2020 was launched in 2002 in Ethiopia. The Federal Ministry of Health has identified 2020 as the target for eliminating blinding trachoma as public health problem in Ethiopia. Moreover, the Amhara region had planned to eliminate blinding trachoma by 2015 which was not yet accomplished [14]. In Amhara region, 10% of the adult population was estimated to have TT. By 2008, 404 health workers had been trained to perform TT surgery in Amhara Region. These individuals are usually stationed in larger health centers where they may perform TT surgery alongside their other duties. Furthermore, outreach surgical campaigns are periodically conducted [8]. However, despite these efforts, surgical services have been relatively low as compared with the need, 125,000 cases operated between 2001 and 2008. Six hundred thousand of non-operated people with TT is estimated in the region [4]. At the study area, the Carter Center’s survey report stated that TT prevalence was 5.90% and the total backlogs were 2,665 of which 1,018 had received the surgical services and the remaining backlogs that didn’t get the service were 1,647 [15]. Fortunately, this study is relevant to support interventions towards clearing the backlogs of TT cases.

In Amhara region, in support of primary eye care services including elimination of blinding trachoma, integrated eye care team was established and members of the team were assigned to play different roles towards TT surgical services. The health cadres assigned as Integrated Eye Care Workers (IECWs) are registered nurses or health officers with additional training to enable them perform trichiasis surgery. They are selected from public health facilities using set criteria as described in the trainees’ manual. They are the ones on whom everyone relies to clear the backlog of TT in the region by delivering surgical services. Program managers are zonal health departments’ and district health offices’ prevention of blindness focal persons, zonal program coordinators, and health centers’ heads. Health care providers are Integrated Eye Care Workers and TT surgery assistants. Health extension workers, kebele leaders and health development army volunteers are support staff. Quality control and monitoring and evaluation roles are given to zonal focal persons, The Carter Center staff (trachoma training and supervisor officers and zonal program coordinators) and mid-level eye care workers. The surgical services are given at static and outreach. This initiative has been working in collaboration to The Carter Center Ethiopia [16].

To reduce trachoma, several countries, including Ethiopia, have made considerable efforts to improve surgical services in the recent years. Unluckily, despite this increased service delivery, the number of operated cases was less than expected. This is due to a range of service and patient-specific barriers [17]. Despite that the magnitude of the problem is high, there is no similar study done in East Amhara sub region. Therefore, it is important to study the determining factors for not utilizing TT surgical services among trachomatous trichiasis patients in district. This study is aimed to identify determinants for not utilizing (inability to use) TT surgery among TT patients in Mehalsayint District, North East Ethiopia, so that strategies will be put in place to overcome the potential barriers.

## Methods

### Study design and setting

This study used a case control study design with patients of untreated trichiasis being cases and those operated being controls. It was conducted in Mehalsayint district, 190 kilometers from Dessie, 675 kilometers East from Bahir Dar and 591 kilometers from Addis Abeba, the capital of Ethiopia. The district had a total population of 83,024 of which 40,848 (49.2%) were men [18]. In the district there are five health centers and fourteen health posts. The TT prevalence of the district was 5.90% and the total backlogs were 2,665 of which 1,018 had received the surgical services and the remaining backlogs that didn’t get the service were 1,647. The District had registered 902 new but not operated TT cases by house to house visits in 2016 [15].

### Source and study populations

All backlogs of the district were the target populations which were 2,665 obtained from Carter Center’s survey report. The study populations were all previously operated and non-operated TT patients registered in the service beneficiary registration book and in TT screened registration book in 2016 respectively living in the district. The previously operated TT patients were 1,018 and considered as controls and the non-operated TT patients were 902 and considered as cases. The study included both controls and cases whose ages were greater than 15 years and registered in 2015/16. Structured and pretested questionnaire was employed for data collection. Data were collected from participants using systematic random sampling technique during March 30, 2017 to April 13, 2017. Trachomatous trichiasis is defined as if at least one eye lash rubbing the eye ball or history of epilation secondary to trachoma [19]. Epilation is operationalized as removing of interned eyelashes mechanically from the eyes by local device (locally known as “*Worento*”) [20].

### Sample size determination and sampling procedure

The sample size was calculated based on two population proportion formula using Epi Info version 7.2. From previous case control studies done in North Ethiopia on determinants of uptake of surgical treatment for trachomatous trichiasis, the major reasons for not using trichiasis surgery by respondents were walking distance and symptoms of interned eye lash. Walking distance from the nearest health facility for < 30 minutes was 33.3% and for >30 minutes was 66.7% with OR of 0.44 (0.19, 0.98). Symptoms of the interned eye lash with persistent pain which was 29.7% and with no persistent pain was 70.3% with OR of 0.57 (0.32, 0.99) [10]. The variables were selected based on the criteria of association, narrow confidence interval, and complete information. The district constitutes a total of five cluster health centers. Each health center encompasses two to five health posts. The operated and non-operated TT cases were registered and the registration was found in the five cluster health centers. Among the total of 1,018 operated and 902 non-operated and registered TT cases, 241 operated (controls) and 241 non-operated (cases) TT cases were selected using systematic random sampling technique from registration lists of each health center. Data were collected from a total of 482 study participants using pretested structured questionnaire by trained data collectors.

### Data analysis

Data were edited, coded and entered into Epi info version 7.2 software packages. It was then exported to Statistical Package for Social Sciences (SPSS) version 20 for analysis. Descriptive analysis was presented using frequency tables, figures, and percentages. In logistic regression, bivariate analysis model was fitted to screen candidate variables with *p-value < 0*.*2* for the final model. To end, multivariable logistic regression analysis model, using backward stepwise method, was employed to identify significant factors for not utilizing TT surgical services. Hosmer and Lemshow goodness of fit test, with *p-value>0*.*05*, was done to test model fitness. Adjusted odds ratio (AOR) with 95% CI and *p-value of <0*.*05* was used to identify independent determinants for not utilizing TT Surgical services.

### Ethical declaration

This study was approved by Ethical Review Committee of College of Medicine and Health Sciences, Wollo University. Written field permits were obtained from South Wollo Zone Health Department and Mehalsayint District Health Office. All study subjects were adults. Verbal/oral consents were taken from the study participants. Since TT cases included in this study will not be stigmatized and even the issue is not sensitive, we preferred oral consents to save time. The consents were taken when interviewing the respondents. In general, it was done with respect to the principles of Declaration of Helsinki.

## Results

### Socio-demographic characteristics

A total of 241 cases and 241 controls of trichiasis were interviewed. Respondents’ mean age was 53.87 (±SD, 14.04) years. Women constitute 153(63.5%) of cases and 149(61.8%) of controls in the study. The majority, 202(83.8%) of cases and 210(87.1%) of controls were unable to read and write. One hundred seventy six (72.6%) of cases and 155(64.3%) of controls were married (Table 1).

**Table 1:** Socio-demographic characteristics of respondents, Mehalsayint district, South Wollo zone, North-east Ethiopia.

Variables	Cases (%) (n = 241)	Controls (%) (n = 241)
**Age in Years**		
16–30	23 (9.5%)	4 (1.7%)
31–45	51 (21.2%)	57 (23.7%)
46–60	100 (41.5%)	101 (41.9%)
>60	67 (27.8%)	79 (32.8%)
**Sex**		
Male	88 (36%)	92 (38.2%)
Female	153 (63.5%)	149 (61.8%)
**Religion**		
Orthodox	235 (97.5%)	215 (89.2%0
Muslim	6 (2.5%)	26 (10.8%)
**Residence**		
Urban	16 (6.6%)	0 (0%)
Rural	225 (93.4%)	241 (100%)
**Marital Status**		
Married	175 (72.6%)	155 (64.3%)
Widowed	36 (14.9%)	50 (20.7%)
Separated/Divorced	21 (8.7%)	28 (11.6%)
Single	9(3.7%)	8(3.3%)
**Education status**		
Unable to read and write	202 (83.8%)	210 (87.1%)
Able to read and write	39 (16.2%)	31 (12.9%)
**Occupation**		
Farmer	192 (79.7%)	241 (100%)
Daily laborer	49 (20.3%)	0 (0%)

### Service related barriers

Among the total respondents, 137(56.8%) of cases and 212(88%) of controls had spent less than or equal to two hours to reach to the surgical services area from their homes. Conversely, 71(29.5%) of cases and 21(8.7%) of controls of the respondents have been reach in greater than two hours walk. One hundred ten (45.6%) of cases faced difficulty of transportation. Twenty three (9.5%) of cases and 3(1.2%) of controls went to surgery sites and returned home by absence of TT surgeon (Table 2).

**Table 2:** Service related barriers for not utilizing trichiasis surgery, Mehalsayint district, South Wollo zone, North-east Ethiopia.

Variables	Cases (%) (n = 241)	Controls (%) (n = 241)
Time to reach to the service	(n = 208)	(n = 233)
< 2 hours	137 (56.8%)	212 (88%)
>2 hours	71 (29.5%)	21 (8.7%)
Difficulty of transportation		
Yes	110 (45.6%)	1 (0.4%)
No	131 (54.4%)	240 (99.6%)
Unavailability of TT surgeon		
Yes	23 (9.5%)	3 (1.2%)
No	218 (90.5%)	238 (98.8%)

### Client/Patient related barriers

Of the total respondents, 86(35.7%) of cases and 124(51.5%) of controls had trichiasis on both eyes. One hundred sixty five (68.5%) of cases and 190(78.8%) of controls were having eye illness for less than five years. Thirty one (12.9%) of cases and five (two percent) of controls had no symptoms of trichiasis. Among the study subjects, 145(60.2%) of cases and 206(85.5%) of controls were practiced epilation. Of which, 93(38.6%) of cases and 142(58.9%) controls for more than one year. Among the study participants, 195(80.9%) of cases and 174(72.2%) of controls have minor trichiasis (≤ 5 lashes). Concerning the outcome of surgery, only 73(30.3%) of cases and 8(3.3%) of controls knew recurrence, whereas 157(65%) of cases and 232(96%) of controls knew good outcome. Almost all of cases and controls are volunteered to get the service if transport costs are covered, even if they get food and shelter service in free of charge for overnight until the dressing is removed (Table 3).

**Table 3:** Client related barriers for not utilizing trichiasis surgery, Mehalsayint district, South Wollo zone, North-east Ethiopia.

Variables	Cases (%) (n = 241)	Controls (%) (n = 241)
Symptoms of trichiasis		
Yes	210 (87.1%)	236 (97.9%)
No	31 (12.9%)	5 (2.1%)
Duration of the problem		
≤ 5 Years	165 (68.5%)	190 (78.8%)
>5 Years	76 (31.5%)	51 (21.2%)
Affected eye		
Right	75 (31.1%)	53 (22%)
Left	80 (33.2%)	64 (26.6%)
Both	86 (35.7%)	124 (51.5%)
Severity of trichiasis		
Minor, ≤ 5 lash	195 (80.9%)	174 (72.2%)
Major, > 5 lash	46 (19.1%)	67 (27.8%)
Epilation practice		
Yes	145 (60.2%)	206 (85.5%)
No	96 (39.8%)	35 (14.5%)
Length of epilation practice	(n = 145)	(n = 206)
≤ 1 Year	52 (21.6%)	64 (26.6%)
>1 Year	93 (38.6%)	142 (58.9%)
Knowing about Trichiasis Surgery		
Yes	205 (85.1%)	233 (96.7%)
No	36 (14.9%)	8 (3.3%)
Place the service given		
Health post	54 (22.4%)	143 (59.3%)
Health Center	154 (63.9%)	90 (37.3%)
I do not know	33 (13.7%)	8 (3.3%)
Volunteer to go to service if transport access		
Yes	238 (98.8%)	241 (100%)
No	3 (1.2%)	0 (0%)
Any person who support you to get service		
Yes	166 (68.9%)	200 (83%)
No	75 (31.1%)	41 (17%)
Knowing a person that undergo TT Surgery		
Yes	179 (74.3%)	233 (96.7%)
No	62 (25.7%)	8 (3.3%)
Outcome of the surgery		
Good	157 (65.1%)	232 (96.3%)
No Improvement	11 (4.6%)	1 (0.4%)
Recurrence	73 (30.3%)	8 (3.3%)
Problem seen after surgery		
Yes	82 (34%)	8 (3.3%)
No	159 (66%)	233 (96.7%)
Known problem that affect surgery		
Yes	32 (13.3%)	0 (0%)
No	209 (86.7%)	241 (100%)
Trust on TT surgeon		
Yes	216 (89.6%)	238 (98.8%)
No	25 (10.4%)	3 (1.2%)
Voluntariness for surgery if shelter & food service		
Yes	240 (99.6%)	239 (99.2%)
No	1 (0.4%)	2 (0.8%)
Participation in discussion session before surgery		
Yes	223 (92.5%)	238 (98.8%)
No	18 (7.5%)	3 (1.2%)
Voluntariness for surgery if get supply		
No	1 (0.4%)	2 (0.8%)
Yes	240 (99.6%)	239 (99.2%)

### Determinants for not utilizing trichiasis surgery

As shown in Table 4 below, being lower age, widowed status, time to reach the service, missed opportunity due to absence of TT surgeon, being unaware of the problem, duration of the problem, the affected eye, epilation practice and lack of information about where the service is given were significant factors of not utilizing the surgical services. The younger (16–30 years) age group was tenfold higher to not get the surgical services as compared to the elders (> 60 years) group (AOR: 10.11, 95% CI: 2.72, 37.59). Respondents that are separated and widowed were 59% and 60% less likely to not get TT surgical services as compared to those that are married (AOR: 0.41, 95% CI: 0.18, 0.94; and AOR: 0.40, 95% CI: 0.21,0. 77) respectively. Respondents who went to surgery site and return by absence of TT surgeon were 5 times more likely not to get TT surgical services than those who did not miss the surgeon (AOR: 5.00, 95% CI: 1.16, 21.38). Respondents who travel < 2 hours walk from their home to reach to the service were 54% less likely to not go to TT surgical services as compared to those who had > 2 hours walk from their home to reach to the service (AOR: 0.46, 95% CI: 0.24, 0.87).

**Table 4:** Determinants for not utilizing trichiasis surgery, Mehalsayint district, South Wollo zone, North-east Ethiopia.

Variables	Cases (n = 241)	Controls (n = 241)	COR (95%CI)		AOR (95% CI)	
			OR (95%CI)	P-Value	OR (95%CI)	P-Value
Age in Years						
16–30	23	4	6.78(2.23,20.58)*	0.001	10.11(2.72, 37.59)**	0.001
31–45	51	57	1.06(0.64, 1.74)	0.833	1.00(0.52, 1.93)	0.990
46–60	100	101	1.17(0.76, 1.79)	0.477	1.07(0.20, 2.44)	0.798
>60	67	79	1.00		1.00	
Marital Status						
Married	175	155	1.00		1.00	
Widowed	36	50	0.64(0.40, 1.03)*	0.066	0.40(0.21,0.77) **	0.006
Divorced	21	28	0.66(0.36, 1.22)	0.186	0.41(0.18,0.94) **	0.034
Single	9	8	1.0(0.38, 2.65)	0.994	0.71(0.20,2.44)	0.581
Time to reach to the service						
≤ 2 hours	137	212	0.19(0.11, 0.33)*	0.00	0.46(0.24, 0.87) **	0.018
>2 hours	71	21	1.00		1.00	
Unavailability of TT surgeon						
Yes	23	3	8.37(2.48, 28.27)*	0.001	5.0(1.16, 21.38)**	0.030
No	218	238	1.00		1.00	
Symptoms of trichiasis						
Yes	210	236	1.00		1.00	
No	31	5	6.97(2.66, 18.25)	0.000	7.49(2.41, 23.26)**	0.001
Duration of the problem						
≤ 5 Years	165	190	1.00		1.00	
>5 Years	76	51	1.72(1.14, 2.59)	0.010	2.56(1.44, 4.54)**	0.001
Affected eye						
Right	75	53	2.04(1.31, 3.19)*	0.002	2.16(1.23, 3.80)**	0.007
Left	80	64	1.80(1.17, 2.77)*	0.007	2.03(1.16, 3.56)**	0.014
Both	86	124	1.00		1.00	
Epilation practice						
Yes	145	206	1.00		1.00	
No	96	35	3.90(2.51, 6.06)*	0.000	3.22(1.84, 5.64)**	0.000
Place the service given						
Health post	54	143	1.00		1.00	
Health Center	154	90	4.53(3.02,6.81)*	0.000	4.21(2.48, 7.14)**	0.000
I do not know	33	8	10.92(4.75,25.14)	0.000	1.01(0.09, 12.10)	0.996

*Significant at bivariate analysis (p<0.2), and

**Significant at multivariable analysis (p<0.05)

Respondents who had not symptoms of trichiasis were 7.49 times more likely to not get TT surgery as compared to those who had symptoms of trichiasis (AOR: 7.49, 95% CI: 2.41, 23.26). Respondents who know the problem for > 5 years were 2.56 times more likely to not go to TT surgical services than those that know the problem for < 5 years (AOR: 2.56, 95% CI: 1.44, 4.54). Respondents who didn’t practice epilation were 3.22 times more likely to not get TT surgical services compared to those who practiced epilation (AOR: 3.22, 95% CI: 1.84, 5.64). Participants whose right eyes with the problem were 2.14 times more likely to not get the TT surgical services than participants whose both eyes affected (AOR: 2.14, 95% CI: 1.23, 3.80). Moreover, participants whose left eyes with the problem were 2.03 times more likely to not get the TT surgical services than participants whose both eyes affected (AOR: 2.03, 95% CI: 1.16, 3.56). Respondents who respond as “the service was given at health center” were 4.21 times more likely to not get TT surgery than those who know the service was given at health post (AOR: 4.21, 95% CI: 2.48, 7.14).

## Discussion

This study identified significant factors for no uptake of TT surgery. The determinants for not use of surgery were respondent’s younger age group and married respondents, long distance from the service, unavailability of TT surgeon, no symptoms of trichiasis, long time knowing the problem, single eye affected, no experience of epilation practice, and they know as “place of service is given at health center".

This finding revealed that the younger respondents were tenfold affected to not get the TT surgical services compared to the elders group (> 60 years). This may be due to doubt by younger age group on the outcomes of surgery for their cosmetics reasons. In addition to the mildness of the condition, they had no severe pain and not to affect their vision. But, as age increases, probably severity of symptoms and disease progresses increase and they go to surgery to alleviate their pain as well as to prevent from suffering from blindness. This finding is in line with a study done in seventeen-outreach campaigns in Amhara Region [21]. In addition to the above reason, lack of time may be common for the young age groups than old patients. Because they may have greater childcare responsibilities, bear much of the responsibility for both agricultural and domestic work and are more likely to be the economically productive members of the family. They may also expect taking more time off work than is necessary after eye lid surgery.

This finding revealed that married participants were more likely to not get the surgical services than their counterparts. This may be due to most married participants mistakenly believed that the surgical wound needs up to 2 months to heal. During this time they should avoid exposure to fire or smoke. Married participants have greater childcare responsibilities, agricultural and domestic work and no body supports the above activity and to prepare food for their family. If they exposed to fire or smoke, they believe the disease recurs. Furthermore, most married women have not the right to take surgery without the willingness of their husband. Attendants who had no symptoms of trichiasis were more likely to not get TT surgical services as compared to their counterparts. This may be due to most respondents in this category had severe form of trichiasis with effect on vision and severe discomfort. Patients with severe discomfort have problem on their vision secondary to the presence of lash on the cornea and the tearing. Such patients consider warranted surgery and want to take the trouble for treatment at present. If most patients reported ‘‘having symptoms”, this may reflect prioritization of eye care over work or farming [10, 21, 22].

This study shows that respondents who knew the problem for short period were better to go to TT surgical services than those that knew the problem for longer periods. This result contradicts with a study done in North Ethiopia, in which the uptake of trichiasis surgery increased with duration of illness [10]. This may be due to mostly the uptake of trichiasis surgery increases when the disease appears new. The uptake of surgery in those with the disease for short duration was good, possibly because severe symptoms might be developed when the disease starts and is not adapted and exposed to other means of local treatment. However, as the disease lasts greater than five years, they habituate the symptom and they utilize local treatment like epilation. The increased uptake of surgical treatment with persistent pain obligates them to deserve attention as the severity of pain is most likely associated with the severity of trichiasis grading and corneal opacity. The trend of seeking surgery at a later stage needs attention in that early surgery is likely to safeguard from troublesome effects.

Respondents who didn’t practice epilation were more likely to not get TT surgical services when compared to those who practiced epilation. This may be due to clients who practice epilation perceived that the disease is severe and utilize surgery as a choice of treatment for long time. They tested as epilation was not permanent treatment and go to TT surgery site to get the service to relief from severe pain and to prevent from blindness. Whereas those respondents who did not practice epilation believe that the disease had no severe pain and did not affect their vision to initiate them for early surgery. Participants whose single eye had the problem were more likely to not get the TT surgical services than those whose both eyes affected. The uptake of surgery in those having bilateral TT was good. This may be due to severity of symptoms increases as the disease attacks both eyes. When both eyes were affected it was indicative of the severity of trichiasis grading and early corneal opacity. This finding is in line with a study done in Tanzania, individuals with bilateral trichiasis were more likely to have surgery than those with unilateral trichiasis [5]. Despite presence of free of charge trichiasis surgical treatment, most of study subjects were using other means of treatment like epilation. This could be due to lack of information, inaccessibility and misconception about trichiasis surgery treatment [22].

This study indicated that respondents who went to surgery site and return by absence of TT surgeon were more likely to not get TT surgery in other times than those who did not miss the Surgeon. This is similar with the study done in Tanzania and Ethiopia [5, 21]. If patients had gone to the health facility for surgery but did not receive the operation due to the surgeon was not present at the time of visit, in such case the patient might lost hope and would not return for surgery. Respondents who said “the service is given at health center” were more likely to not get TT surgery than those who knew the service is given at health post. Surgical campaigns in health posts may be particularly effective at reaching larger numbers of patients in Ethiopia, though feasibility issues challenge the service delivery as it is a large country with very limited infrastructure and trained personnel [17, 23]. Moreover, a walking distance to the service area for greater than an hour showed an association with decreased attendance for TT surgery in Amhara region [10]. Surgical campaigns in health posts are also likely to be particularly beneficial for reaching women and older people for whom transport, distance and lack of attendant were particular barriers [21].

The study shows that patients who live in the nearby health facility were better to have had surgery as compared to those that are distant. This is similar with a study done in North Ethiopia [10]. It may be due to those trichiasis patients even with the presence of post-surgical eye packs travel easily to their home after getting the service, which indicates as the service is near their home, awareness of utilizing the existing service improves. Our study is strong in that it provided operational recommendations based on the identified factors. However, it is limited in which the study uses TT cases that have been already screened by Health Extension Workers (HEWs) and Integrated Eye Care Workers (IECWs) and has not tried to search new cases because of cost and time. No follow up were conducted to ensure whether or not the patients have gone to the health facilities to get the surgical services based on the advice given to them when interviewed. Consequently, it may affect the result and recommendations given here. Prospective study is recommended to identify the root causes for not uptake of surgery treatment.

The findings of this study have valuable policy implications for health programs scheme and interventions. Training and deploying of HEWs and Community-based screeners on house to house screening and awareness creation activities; expanding the service to health post level with campaign and outreach services; and posting the service days on different places may be important to reduce the problem. In other words, this findings supreme important to Mehalsayint district health office and the respective zonal health department, and partners to develop interventions programs against this neglected tropical disease.
